# Recovering Scene Geometry and Material from Event Streams with 3D Gaussian Splatting

**DOI:** 10.3390/s26175584

**Published:** 2026-09-02

**Authors:** Zehao Chen, Binbin Zhou, Zengwei Zheng

**Affiliations:** 1 School of Computer and Computing Science, Hangzhou City University, Hangzhou 310015, China; 2 College of Computer Science and Technology, Zhejiang University, Hangzhou 310027, China

**Keywords:** event camera, 3D Gaussian splatting, inverse rendering, scene decomposition

## Abstract

Recovering scene geometry and material yields physically meaningful representations that support many tasks in computer vision and graphics. Methods built on neural representations such as neural radiance fields and 3D Gaussian Splatting perform well but assume clean, well-exposed multi-view images, and their accuracy degrades substantially under low light, fast motion, or strong illumination changes. We instead recover scene geometry and material using only event streams (the asynchronous output of a neuromorphic event camera, which records per-pixel logarithmic-brightness changes above a contrast threshold) as input, within a framework based on 3D Gaussian Splatting. Because events measure differential brightness and leave the global brightness scale undetermined, we introduce a brightness-anchoring prior that constrains this global brightness scale while reconstructing a radiance field; we then decompose the radiance into geometry and material (surface normals, depth, base color, and roughness), using motion-compensated event edges to guide boundary-aware regularization. On public datasets, the method achieves the best average performance among baselines that first reconstruct images and then apply inverse rendering. Averaged over the seven evaluated scenes, it attains 22.11 dB PSNR, 0.902 SSIM, and 0.105 LPIPS on novel-view rendering, and 22.31 dB PSNR on base-color estimation, with a mean surface-normal angular error of $28.93^{\circ }$. The quantitative evaluation uses simulated event streams on seven synthetic scenes; we additionally include a qualitative demonstration on a physical event-camera capture, and identify full quantitative validation on real recordings as the primary direction for future work.

## 1. Introduction

Recovering scene geometry and material is a core problem in computer vision and graphics: an interpretable, physically grounded description of a scene supports downstream tasks such as scene understanding, appearance editing, and relighting [1,2]. Neural scene representations such as neural radiance fields (NeRFs) [3] and 3D Gaussian Splatting (3DGS) [4] have pushed 3D reconstruction and novel-view synthesis to high quality. They have also opened the door to decomposing a scene into explicit geometry and material [1,5], so that the recovered representation is realistic to render as well as physically meaningful and editable [6,7,8,9,10,11].

NeRF/3DGS approaches typically optimize scene representations from multi-view RGB observations, where accurate multi-view relations, colors, and gradients are essential for recovering scene geometry and material [12]. However, such requirements are often difficult to satisfy in real-world settings. In practical scenarios such as hand-held low-light capture, nighttime imaging, or scenes involving fast motion and strong illumination variation, images can be severely degraded by low visibility, sensor noise, motion blur, saturation, and high-dynamic-range effects. As a result, these cues often become inaccurate or unstable, leading to degraded decomposition performance.

**Figure 1:**
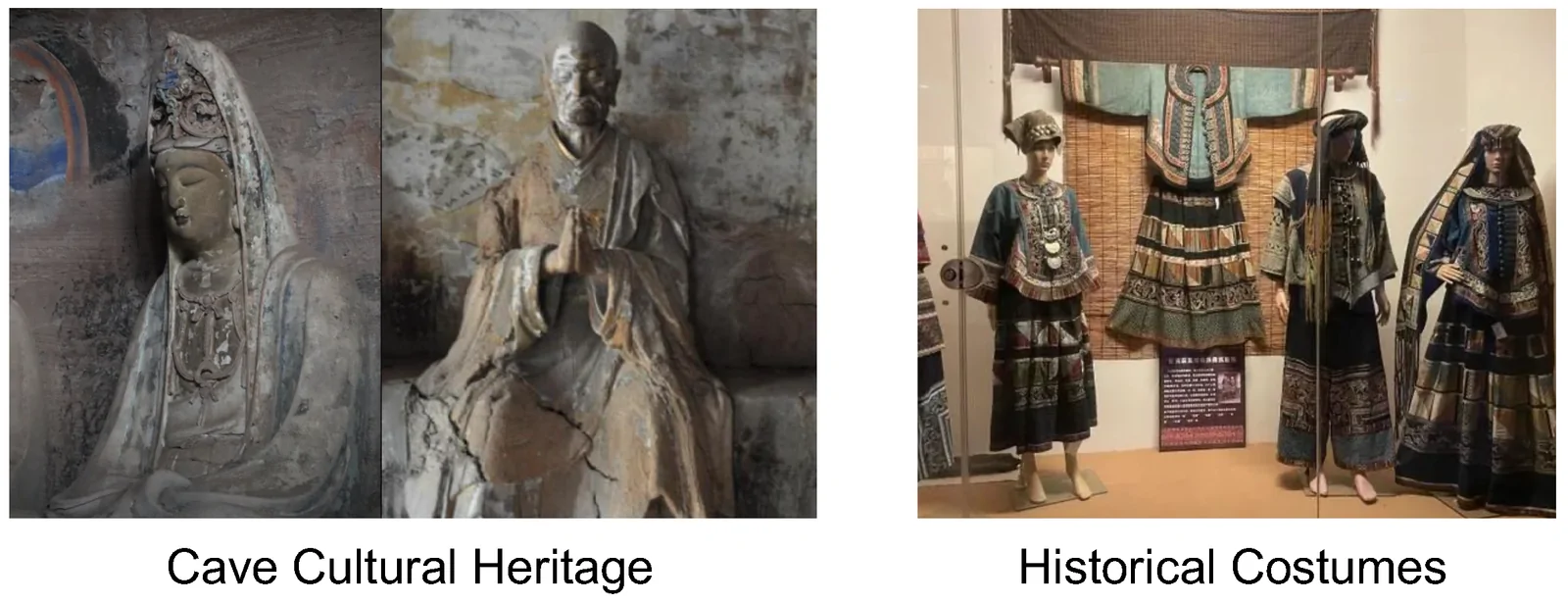
Two cultural-heritage capture scenarios in which conventional acquisition conditions cannot be freely controlled: (**left**) polychrome sculptures and wall paintings preserved in a dim cave environment (illumination constraint), and (**right**) fragile historical costumes exhibited in a museum (object-handling constraint). The figure is included as application motivation, not as experimental validation.

Such difficulties are common in cultural-heritage digitization, where conventional acquisition conditions cannot be freely controlled (Figure 1). Cave sculptures and wall paintings are commonly preserved under limited ambient illumination; although stronger artificial lighting could improve frame-based image quality, accumulated optical exposure may induce fading or darkening in light-sensitive pigments and therefore needs to be carefully controlled [13,14], so during moving-camera acquisition, conventional cameras face a trade-off between illumination intensity, exposure time, sensor noise, and motion blur. Historical costumes present a different constraint: their fragile and deformable materials restrict handling and repositioning, making it difficult to maintain a stable configuration throughout multi-view capture [15]. These examples show that careful studio preparation is not always feasible, and event cameras, owing to their high temporal resolution and high dynamic range, provide a potential sensing modality when illumination and camera motion cannot be fully controlled; we do not claim that event sensing resolves non-rigid deformation, but it may alleviate the imaging degradation caused by restricted illumination and camera motion.

Event cameras, which asynchronously record brightness changes with high temporal resolution and high dynamic range, have been increasingly incorporated into scene reconstruction [16,17,18] and decomposition and lighting estimation [5,19] as useful auxiliary cues under such challenging conditions. However, methods that combine events with images still rely on RGB observations as the anchor for appearance modeling, and thus remain sensitive to image quality. Capturing both modalities also requires a hybrid or beam-splitter camera rig with careful spatial and temporal calibration [20,21,22], which complicates deployment.

We ask whether scene geometry and material can be recovered from events alone, without any RGB image. Two difficulties make the problem ill-posed: the event stream is sparse and asynchronous, and it does not determine absolute intensity or color, which is precisely what material and color estimation depend on. One workaround first reconstructs images from events with event-to-video methods [23,24] and then runs a standard image-based inverse-rendering pipeline. However, the reconstructed images are not optimized to preserve the multi-view consistency, colors, and gradients that reliable decomposition relies on, so reconstruction artifacts propagate into the result.

Two properties of event data make a direct approach feasible. Events measure differential changes in scene radiance, which lets us relate them to geometry and material through a radiance field, and they carry edge information [25] that helps locate material and geometry boundaries.

**Figure 2:**
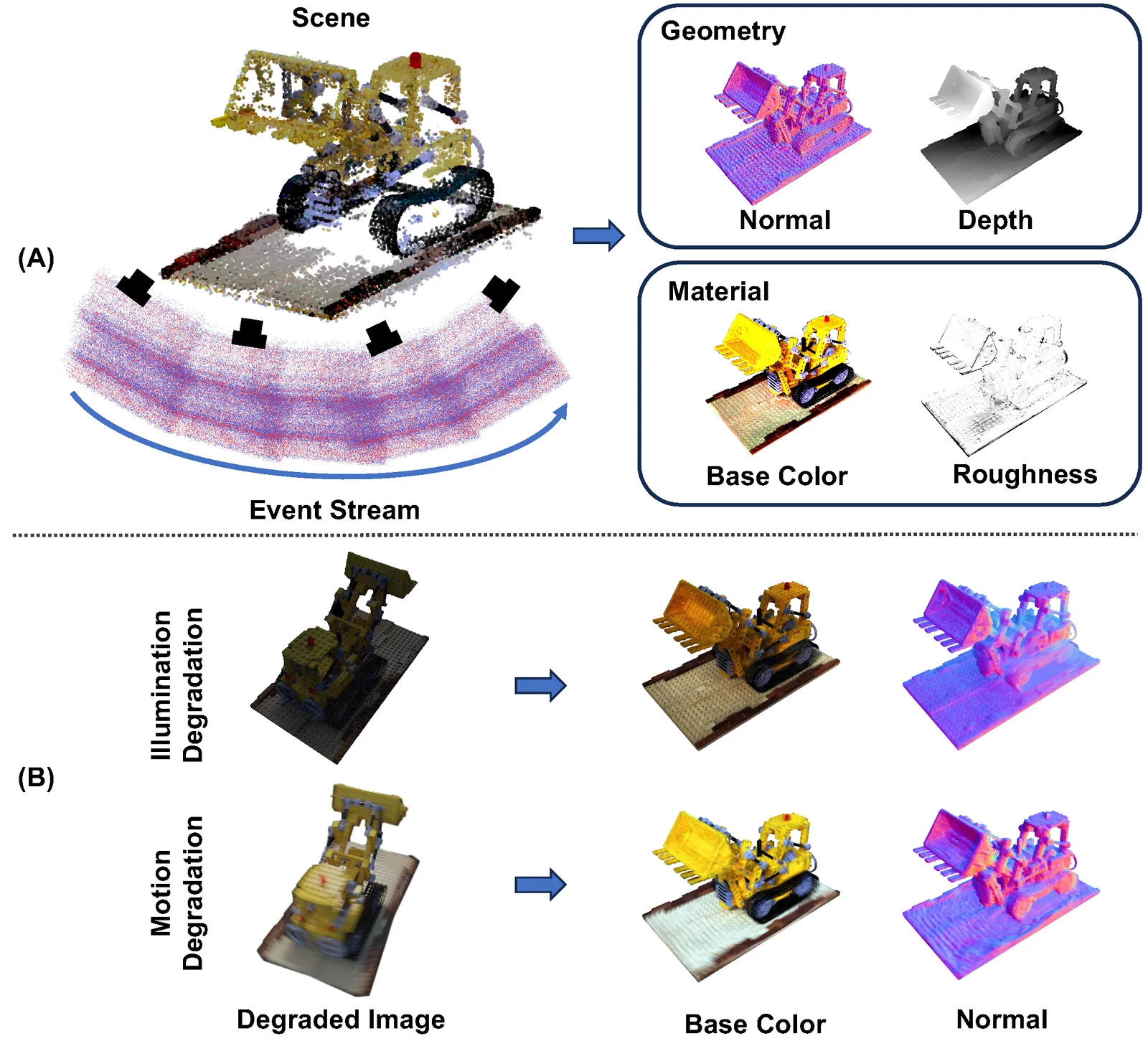
Overview of our event-only scene decomposition. (**A**) Taking only an event stream as input, without any RGB image, our method constructs a radiance field and decomposes the scene into geometry (surface normals and depth) and material (base color and roughness). (**B**) Robustness under degraded conditions: Under illumination degradation (low-light) and motion degradation (motion blur), the captured images are severely corrupted, so image-based decomposition methods fail, whereas our method still recovers base color and normals directly from events.

We use both properties in a 3DGS-based framework that decomposes scene geometry and material directly from events (Figure 2). It reconstructs a radiance field, in which a brightness-anchoring prior constrains the underdetermined global brightness scale, and then decomposes this radiance into geometry and material, using motion-compensated event edges to guide boundary-aware regularization. On public datasets, decomposing directly from events achieves the best average performance compared with baselines that first reconstruct images and then invert them.

The main contributions can be summarized as follows:

- We introduce event-only scene geometry and material decomposition: a 3D Gaussian Splatting framework that recovers geometry and material from event streams alone, without any image input. The recovered material representation is constrained: base color is recovered reliably, whereas roughness is only weakly constrained and metallicity is fixed under a dielectric assumption, so we do not claim general recovery of spatially varying physically based material parameters.
- We formulate an event-domain reflectance model that links the event generation process to physically based scene radiance and material, and that accounts for occlusion and indirect illumination in multi-object scenes.
- We derive two priors from the events themselves: a brightness-anchoring prior that constrains the underdetermined global brightness scale, and an event-derived structural cue that uses motion-compensated event edges for boundary-aware regularization.

We evaluate the proposed framework on simulated event streams generated from synthetic multi-view scenes, which provides a controlled quantitative evaluation; we additionally include a qualitative demonstration on a physical event-camera capture and identify full quantitative validation on real event sensors as the primary direction for future work. The framework assumes known camera poses and intrinsics.

## 2. Related Work

The existing studies that are most related to ours fall into three categories. Image-based scene-decomposition methods estimate geometry, material, and illumination from multi-view RGB images. Event-guided decomposition methods use events together with conventional images. Event-based reconstruction methods recover geometry and radiance from event streams but do not decompose the reconstructed appearance into material and illumination. In contrast, our method uses event streams as the sole captured appearance observation and retains event-domain constraints during both geometry reconstruction and material decomposition.

### 2.1. Scene Geometry and Material Decomposition from Multi-View Images

Decomposing multi-view images into explicit geometry and material is a well-studied problem in computer vision and graphics. Neural scene representations have advanced it considerably by embedding physically based rendering into differentiable pipelines.

Along the NeRF line, NeRFactor [7] pioneered the factorization of shape, reflectance, and illumination within a neural radiance field, recovering surface normals, base color, and BRDFs from multi-view images under unknown lighting. PhySG [8] modeled illumination with spherical Gaussians to enable physics-based material editing and relighting, though its smooth lighting representation limits the recovery of fine-grained material details. TensoIR [11] leveraged the efficient voxel grid structure of TensoRF to accelerate BRDF estimation and explicitly modeled visibility and indirect illumination for improved decomposition quality. IntrinsicNeRF [6] introduced intrinsic decomposition into the NeRF framework, factorizing scenes into reflectance, shading, and residual layers to support editable novel-view synthesis on room-scale scenes. NVDiffRec [9] adopted a differentiable rendering pipeline to jointly extract triangular meshes, spatially varying materials, and environment lighting from multi-view observations.

More recently, 3D Gaussian Splatting (3DGS) [4] has emerged as an efficient alternative to NeRF, enabling real-time rendering with explicit scene representations. GS-IR [1] was among the first to extend 3DGS for inverse rendering, proposing depth-derivation-based normal estimation and baking-based occlusion modeling for indirect illumination. GShader [10] augmented 3D Gaussians with shading functions to better handle reflective surfaces, introducing a normal estimation strategy based on the shortest axis of Gaussian ellipsoids.

All of these methods, however, take high-quality multi-view RGB images as input, relying on accurate photometry, multi-view consistency, and image gradients to recover geometry and material [1,11,12]. When the images are degraded by low light, fast motion, sensor noise, or high dynamic range, these cues become unreliable and the decomposition suffers, which is why we turn to a sensing modality that stays robust under these conditions.

### 2.2. Event-Based 3D Reconstruction and Material Estimation

Event cameras asynchronously record per-pixel brightness changes with microsecond latency and high dynamic range (140 dB) [21], which makes them well suited to conditions where frame-based cameras fail. This has prompted a growing body of work that uses event streams for 3D scene understanding, most of it aimed at geometric reconstruction.

Several recent works have incorporated event data into neural radiance field frameworks to improve 3D geometry reconstruction under adverse conditions. E2NeRF [18] proposed a unified framework that jointly learns from blurry images and event streams to reconstruct sharp NeRFs, showing that combining the two modalities benefits geometry recovery. EvHDR-NeRF [17] and EvHDR-GS [16] exploited the high dynamic range of event cameras to enable HDR novel-view synthesis under extreme lighting conditions, achieving improved geometric reconstruction in over-exposed and under-exposed regions. These methods, however, target geometry and appearance only, and do not decompose material properties such as base color, roughness, or surface normals.

EventID [5] represents the first attempt to incorporate event data into the material estimation pipeline, leveraging event streams as auxiliary cues to assist image-based inverse rendering. However, EventID still relies on RGB images as the primary input for appearance modeling and material estimation, with event data serving only as a supplementary signal. As a result, its performance remains sensitive to image quality, and the requirement for jointly capturing both modalities necessitates hybrid camera systems [20,21,22], which limits practical deployment flexibility.

In summary, existing event-based methods primarily focus on geometric reconstruction, while the only work addressing material estimation remains dependent on RGB input. Recovering geometry and material from event streams alone, with no image input, remains largely unexplored. To the best of our knowledge, this is the first event-only framework for scene geometry and material decomposition based on 3D Gaussian Splatting; it links event observations to scene geometry and material through a radiance field and two event-derived priors.

### 2.3. Event-Guided Reconstruction Under Challenging Imaging Conditions

Event cameras are particularly attractive in imaging conditions involving rapid motion, high dynamic range, or limited illumination. Jiang et al. [26] introduced an event-guided motion-deblurring framework that uses the high temporal resolution of events to recover sharp image sequences from motion-blurred observations. Beyond two-dimensional image restoration, several studies have incorporated event measurements into neural scene representations. Ev-NeRF [27] reconstructs radiance fields from event measurements under extreme noise and high-dynamic-range conditions, while EventNeRF [28] demonstrates event-only novel-view synthesis using a color event camera, including experiments involving fast motion and low illumination. Robust e-NeRF [29] further considers sparse and noisy events under non-uniform camera motion and models several event-sensor non-idealities. E2NeRF [18] combines events with motion-blurred RGB images to reconstruct sharp radiance fields in complex and low-light scenes. These methods establish the value of event measurements for image restoration and radiance-field reconstruction under challenging imaging conditions. In contrast, our work focuses on decomposing the recovered scene representation into geometry, reflectance, and illumination without using RGB images as appearance input.

## 3. Background

### 3.1. 3D Gaussian Splatting

3D Gaussian Splatting (3DGS) [4] represents a scene as a collection of 3D Gaussian primitives $G={\left\{G_{i}\right\}}_{i=1}^{M}$. Each Gaussian $G_{i}$ is parameterized by its center position $\mu_{i}\in R^{3}$, a 3D covariance matrix $\Sigma_{i}$ (represented via a scaling vector $s_{i}$ and a rotation quaternion $q_{i}$), an opacity $\alpha_{i}$, and a set of spherical harmonics (SH) coefficients $f_{i}$ encoding view-dependent color.

For rendering, the 3D Gaussians are projected onto the image plane via the viewing transformation, yielding 2D Gaussians. The color $\hat{C}\left(r\right)$ at a pixel r is computed by alpha-compositing the contributions of all overlapping Gaussians sorted by depth:

(1) $$\hat{C}\left(r\right)=\sum_{i\in N}c_{i}\;\alpha_{i}^{\prime }\prod_{j=1}^{i-1}\left(1-\alpha_{j}^{\prime }\right),$$

where $c_{i}$ is the color of Gaussian $G_{i}$ evaluated from its SH coefficients, $\alpha_{i}^{\prime }$ is the effective opacity obtained by multiplying the learned opacity $\alpha_{i}$ with the 2D Gaussian evaluated at pixel r, and N denotes the set of Gaussians overlapping the pixel.

### 3.2. Event Camera

An event camera is a bio-inspired sensor that asynchronously measures per-pixel brightness changes [21]. Unlike conventional frame-based cameras that capture dense intensity images at fixed intervals, each pixel of an event camera independently monitors the log intensity and triggers an event whenever the change exceeds a predefined contrast threshold. The output is an asynchronous stream of events $E={\left\{e_{k}\right\}}_{k=1}^{N}$, where each event $e_{k}=\left(x_{k},y_{k},t_{k},p_{k}\right)$ records the pixel location $\left(x_{k},y_{k}\right)$, timestamp $t_{k}$, and polarity $p_{k}\in \left\{+1,-1\right\}$ indicating the direction of brightness change. Event cameras offer high temporal resolution (on the order of microseconds), high dynamic range (∼140 dB), and low power consumption, making them particularly advantageous under conditions involving fast motion, low light, and extreme illumination variation. The color-event model used in our experiments follows an RGGB Bayer color-filter array, a configuration adopted in several color-event imaging and reconstruction studies [28,30]. Each pixel measures temporal contrast in a single color channel determined by its position in the Bayer pattern, so the event stream provides channel-dependent relative radiance changes rather than a direct measurement of absolute RGB intensity.

## 4. Proposed Method

Given a set of event streams E captured by a moving event camera with known camera poses, our goal is to recover geometry, represented by surface normals and depth, together with material parameters (base color, roughness, and metallicity), without any RGB image input. The camera poses and intrinsics are treated as known geometric inputs. They need not be obtained from conventional RGB images: they can be recovered from the events themselves, either by converting the event stream into an intensity video with an event-to-video method [30] and running a standard structure-from-motion pipeline such as COLMAP [31], or by a pose-free event method that jointly estimates poses and scene from a single event camera, such as IncEventGS [32]. A full evaluation with event-derived poses is left to future work; the present contribution is the decomposition given known poses. We use the term “event-only” to refer to appearance supervision: the event stream is the only captured appearance observation used to constrain scene radiance and material properties. Besides the event stream, the framework uses camera poses and intrinsics; it does not use conventional RGB images, depth maps, object masks, scene bounds, or an RGB-derived structure-from-motion point cloud, and the Gaussian representation is initialized from random points.

Our method consists of three key components: (i) an event-domain scene decomposition model that establishes the relationship between event observations and scene geometry/material under the Lambertian assumption (Section 4.1); (ii) two priors, a brightness-anchoring strategy and event-derived gradient cues, that compensate for the missing appearance information in event streams (Section 4.2); and (iii) a two-stage optimization framework that first reconstructs scene geometry via a radiance field and then performs material decomposition (Section 4.3).

### 4.1. Event-Based Reflectance Model

The existing event-based reflectance model [5] is designed for single isolated objects, where only direct illumination from the environment is considered. However, in real-world scenes containing multiple objects, inter-object occlusion [1] and indirect illumination [11,33] play a significant role in determining the observed radiance. Ignoring these effects leads to systematic decomposition errors such as shadow–base-color entanglement [6] and incorrect material attribution from color bleeding [11]. We propose an event-based reflectance model that extends the formulation to account for occlusion and indirect illumination, enabling geometry and material decomposition in complex scenes.

An event $e_{k}$ is triggered at pixel $\left(x_{k},y_{k}\right)$ when the log-radiance change since the last event exceeds a contrast threshold *C*:

(2) $$p_{k}(\log L\left(x_{k},y_{k},t_{k}\right)-\log L\left(x_{k},y_{k},t_{k}-\Delta t_{k}\right))=C,$$

where L(x,y,t) denotes the intensity at pixel (x,y) and time *t*, and $\Delta t_{k}$ is the time elapsed since the last event at the same pixel.

The radiance *L* observed at each pixel is determined by the physical interaction between illumination, scene geometry, and surface material. In complex scenes, the outgoing radiance at a surface point x with normal n observed from direction $\omega_{o}$ consists of direct and indirect components:

(3) $$L\left(x,\omega_{o}\right)=L_{\mathrm{dir}}\left(x,\omega_{o}\right)+L_{\mathrm{ind}}\left(x\right),$$

where $L_{\mathrm{dir}}$ is the direct illumination from the environment and $L_{\mathrm{ind}}$ is the indirect illumination from inter-object light transport. The existing single-object model [5] assumes unoccluded illumination and omits the indirect term; recovering material correctly in multi-object scenes requires modeling both explicitly, which we detail next.

The direct illumination term is given by

(4) $$L_{\mathrm{dir}}\left(x,\omega_{o}\right)=\int_{\Omega^{+}}V\left(x,\omega_{i}\right)\;L_{\mathrm{env}}\left(\omega_{i}\right)\;f_{r}\left(x,\omega_{i},\omega_{o}\right)\;\left(\omega_{i}\cdot n\right)\;d\omega_{i},$$

where $L_{\mathrm{env}}\left(\omega_{i}\right)$ is the environment illumination; $f_{r}$ is the Cook–Torrance BRDF [34] parameterized by base color a, roughness *r*, and metallic *m*; and $V\left(x,\omega_{i}\right)\in \left[0,1\right]$ is the visibility function that encodes whether the incident direction $\omega_{i}$ at point x is occluded by other objects in the scene. In single-object settings, V≡1 since no other geometry blocks the environment light. In scenes, neighboring objects cast shadows and block portions of the incident hemisphere, making *V* spatially varying and direction-dependent. Without explicit visibility modeling, shadow effects are incorrectly absorbed into the estimated base color, a common failure mode known as shadow–base-color entanglement.

The indirect illumination term $L_{\mathrm{ind}}\left(x\right)$ captures light reflected by other surfaces before reaching point x, accounting for inter-object light transport such as color bleeding, where a brightly colored object tints the appearance of nearby surfaces.Without this term, such illumination effects corrupt the material decomposition by being absorbed into the estimated base color.

### 4.2. Event-Derived Cues for Scene Decomposition

Existing scene decomposition methods [1,11,33] rely on dense RGB images, where per-pixel color and spatial gradients directly provide appearance and structural cues for separating material from illumination. In contrast, the event stream is essentially a spatio-temporal point cloud: each event records only a pixel location, a timestamp, and a polarity, carrying no absolute brightness or color information. This fundamental difference poses unique challenges for material decomposition. To bridge this gap, we introduce two constraints derived from the events. The first is a brightness-anchoring prior that constrains the underdetermined global brightness scale of the reconstructed radiance field. The second is an event-derived structural cue that uses motion-compensated event edges to guide boundary-aware regularization.

#### 4.2.1. Global Brightness Anchoring

The event generation model (Equation (2)) records only the differencein log-radiance between consecutive observations at each pixel, leaving the absolute brightness and color of the scene unobservable. Event measurements therefore constrain temporal changes in log radiance but do not provide an absolute radiance reference. We introduce a global brightness anchor that constrains the scalar mean brightness of the rendered training observations to remain close to a fixed target g=0.5. This fixed value provides a common brightness normalization for all scenes and is not inferred from ground-truth RGB images or material maps; the anchor does not impose an achromatic mean scene color or independently modify the relative red, green, and blue channel balance:

(5) $$L_{\mathrm{GW}}={\left|\frac{1}{\left|R\right|}\sum_{r\in R}\frac{{\hat{C}}_{R}\left(r\right)+{\hat{C}}_{G}\left(r\right)+{\hat{C}}_{B}\left(r\right)}{3}-g\right|}^{2},$$

where R is the set of rendered pixels over the training observations, ${\hat{C}}_{R},{\hat{C}}_{G},$ and ${\hat{C}}_{B}$ are the rendered color channels, and g=0.5 is the fixed target brightness. The earlier vector form of this loss, written as a constraint on the mean RGB color, was a typographical error in the loss expression; the implementation used in all experiments computes the scalar mean rendered brightness as above, so this correction does not affect the reported results. The target g=0.5 is a fixed brightness normalization rather than a physical radiometric calibration: the absolute brightness of the reconstructed appearance should be interpreted relative to this normalization, and applications requiring absolute radiance measurements may replace the fixed target with an externally calibrated reference. Moreover, because the event generation model responds to temporal changes of the logarithmic radiance, it is invariant to an independent global multiplicative factor applied to each color channel; these three per-channel factors form a gauge ambiguity that events alone cannot resolve. The RGGB events constrain the spatial and temporal variation within each channel, that is, the relative color pattern, up to one unknown global constant per channel, whereas the anchor above imposes only a single scalar constraint on the mean intensity. This fixes the overall luminance scale but not the two remaining inter-channel ratios, so the absolute global color balance is not uniquely identifiable from events alone and, when required, must be fixed by an external reference.

#### 4.2.2. Structural Cues from Events

Under camera motion, events tend to concentrate around strong image gradients, which often coincide with object boundaries, depth discontinuities, and material transitions [25,26]. Accumulating events within a short temporal slice therefore yields an edge map that reflects much of the structural layout of the scene. Since the event density is inherently coupled with camera motion, a naive fixed-duration slice produces streak artifacts and fails to reflect the true spatial gradient information; we therefore adopt contrast maximization [35] to perform motion-compensated event accumulation, yielding sharp edge maps that better approximate the underlying image-gradient structure of the scene. Formally, given the events $\left\{e_{k}=\left(x_{k},t_{k},p_{k}\right)\right\}$ within a temporal slice $E_{s}$, we warp each event to a common reference time $t_{\mathrm{ref}}$ and accumulate the warped events into the edge map

(6) $$M_{\mathrm{edge}}\left(r\right)=\frac{1}{Z}\sum_{e_{k}\in E_{s}}\delta \;\left(r-x_{k}^{\prime }\right),\;x_{k}^{\prime }=x_{k}-\left(t_{k}-t_{\mathrm{ref}}\right)\;v\left(x_{k}\right),$$

where $x_{k}^{\prime }$ is the motion-compensated position of event $e_{k}$ under the optical flow v, and *Z* normalizes $M_{\mathrm{edge}}$ to [0,1]. The flow v is estimated by maximizing the contrast (variance) of the accumulated map, so that events triggered by the same edge align into a sharp response rather than a motion-induced streak.

We use this event-derived edge map $M_{\mathrm{edge}}$ to guide a total variation (TV) regularization on the decomposition outputs. A standard TV loss penalizes the spatial gradients of the predicted maps to suppress high-frequency artifacts, but a spatially uniform TV also over-smooths genuine boundaries. Because these events align with likely discontinuities, the edge map provides an indication of where discontinuities are likely to occur and should be preserved. We therefore down-weight the TV penalty at event locations, yielding an event-guided TV loss:

(7) $$L_{\mathrm{TV}}=\sum_{r}w\left(r\right)\left(∥\nabla \hat{a}{\left(r\right)∥}_{1}+\lambda_{n}{∥\nabla \hat{n}\left(r\right)∥}_{1}\right),$$

where ∇ denotes the spatial gradient operator, $\hat{a}$ and $\hat{n}$ are the rendered base color and normal maps, and the weight $w\left(r\right)=\exp(-\beta \cdot M_{\mathrm{edge}}\left(r\right))$ decreases with the local event density. At edge locations, where events are dense, the small weight relaxes the TV penalty and permits sharp transitions in base color and normals; in smooth regions, where few events fire, the large weight enforces strong TV smoothness, preventing high-frequency artifacts in the decomposition.

### 4.3. Framework

We implement the scene-level event-based reflectance model (Section 4.1) within the 3D Gaussian Splatting framework. Since events are differential signals of radiance, we treat radiance as the intermediate representation that bridges event observations and material properties. Jointly optimizing geometry and material from events alone is ill-posed: events carry no absolute appearance, and the two sets of unknowns are tightly coupled. We therefore split the problem into two stages: Stage I reconstructs the scene geometry and a view-dependent radiance field from event streams, and Stage II decomposes this radiance into material properties using the physically based reflectance model.

#### 4.3.1. 3DGS-Based Scene Representation

We adopt 3DGS as the scene representation. Beyond the standard Gaussian attributes, each Gaussian $G_{i}$ is augmented with appearance properties that differ between the two stages: in Stage I, each Gaussian stores SH color coefficients $f_{i}$ for view-dependent radiance; in Stage II, they are replaced by material parameters (base color $a_{i}$, roughness $r_{i}$, metallic $m_{i}$), baked visibility SH $V_{i}$, and learnable indirect lighting SH $L_{\mathrm{ind},i}$, with the radiance computed via the physically based rendering model (Equation (3)). The per-pixel radiance $\hat{L}$ at pixel r from viewing direction $\omega_{o}$ is obtained by alpha-compositing over the contributing Gaussians:

(8) $$\hat{L}\left(r,\omega_{o}\right)=\sum_{i\in N}L\left(x_{i},\omega_{o}\right)\;\alpha_{i}^{\prime }\prod_{j=1}^{i-1}\left(1-\alpha_{j}^{\prime }\right),$$

where $L(x_{i},\omega_{o})$ is the outgoing radiance at Gaussian $G_{i}$.

#### 4.3.2. Optimization Strategy

We optimize the framework in two stages that separate geometry reconstruction from material decomposition. In Stage I, we reconstruct the scene geometry and a view-dependent radiance field purely from event streams. The brightness-anchoring prior (Section 4.2) constrains the underdetermined global brightness scale, stabilizing the radiance field when absolute brightness is unavailable. This stage yields accurate surface geometry together with a radiance field that serves as the color cue for the subsequent decomposition.

In Stage II, we fix the geometry and decompose the recovered radiance into material properties, assigning each Gaussian learnable base color, roughness, and metallic parameters under the reflectance model of Equation (3), which accounts for both direct and indirect illumination. The environment illumination is jointly estimated with the material parameters. The decomposition is driven by three signals: the radiance from Stage I provides color cues, the event-domain loss enforces consistency with the observed events, and the event-derived structural cue (Section 4.2) guides boundary-aware regularization.

#### 4.3.3. Loss Functions

The event-domain loss is the core supervision signal throughout both stages, derived from the event generation model (Equation (2)). For each pixel r, we accumulate the events triggered within a short time interval [t−Δt,t] and match the resulting integrated log-intensity change to the change of the rendered radiance over the same interval:

(9) $$L_{\mathrm{event}}=\sum_{r}{∥\;C\;\;\;\sum_{k\in N(r,\;t-\Delta t,\;t)}\;\;\;p_{k}\;-\;\left(\log\hat{L}\left(r,\omega_{o}^{t}\right)-\log\hat{L}\left(r,\omega_{o}^{t-\Delta t}\right)\right)∥}_{1},$$

where N(r,t−Δt,t) denotes the set of events triggered at pixel r within the interval [t−Δt,t], so that $C\sum_{k}p_{k}$ is the accumulated log-intensity change measured by the events, and the second term is the corresponding log-radiance difference predicted by the rendered radiance field between the two time instants. For color events, the rendered quantity $\hat{L}\left(r,\cdot \right)$ is the single rendered radiance channel, red, green, or blue, selected according to the RGGB Bayer position of pixel r, so the loss compares the accumulated signed events with the temporal log-radiance change of the matching color channel. Spatial and channel-dependent color variations are thereby constrained directly by the Bayer-sampled event observations, whereas only the global brightness scale is left underdetermined and fixed by the global brightness anchor (Equation (5)). When no event is recorded at a pixel during an interval, the accumulated event count $C\sum_{k}p_{k}$ is zero; this value acts as an optimization surrogate that encourages the rendered log-radiance change to remain small, and is not interpreted as a physical measurement that the true radiance change is exactly zero. Moreover, because the scene is a shared set of 3D Gaussians observed across many intervals and viewpoints, a location that produces no event in one interval is still constrained by the events it triggers in other observations, so an inactive pixel in a single interval does not independently determine the corresponding scene point. In Stage I, the training objective is

(10) $$L_{\mathrm{stage}1}=L_{\mathrm{event}}+\lambda_{\mathrm{GW}}L_{\mathrm{GW}}+\lambda_{\mathrm{normal}}L_{\mathrm{normal}},$$

where $L_{\mathrm{GW}}$ is the brightness-anchoring loss (Section 4.2). The normal loss keeps the rendered normals consistent with the geometry implied by the rendered depth,

(11) $$L_{\mathrm{normal}}=\sum_{r}{∥\hat{n}\left(r\right)-n_{d}\left(r\right)∥}_{1},$$

where $\hat{n}\left(r\right)$ is the rendered normal at pixel r and $n_{d}\left(r\right)$ is the pseudo-normal computed from the local gradients of the rendered depth. In Stage II, the training objective is

(12) $$L_{\mathrm{stage}2}=L_{\mathrm{event}}+\lambda_{\mathrm{rad}}L_{\mathrm{rad}}+\lambda_{\mathrm{GW}}L_{\mathrm{GW}}+\lambda_{\mathrm{TV}}L_{\mathrm{TV}},$$

where $L_{\mathrm{TV}}$ is the event-guided total variation regularization (Equation (7), Section 4.2), and $L_{\mathrm{rad}}$ supervises the physically based rendered radiance against the radiance recovered in Stage I:

(13) $$L_{\mathrm{rad}}=\sum_{r}{∥{\hat{L}}_{\mathrm{pbr}}\left(r,\omega_{o}\right)-{\hat{L}}_{S1}\left(r,\omega_{o}\right)∥}_{1},$$

where ${\hat{L}}_{\mathrm{pbr}}\left(r,\omega_{o}\right)$ is the radiance obtained by physically based rendering from the recovered material and illumination (Section 4.1), and ${\hat{L}}_{S1}\left(r,\omega_{o}\right)$ is the radiance rendered by the frozen Stage-I radiance field, which provides a stable per-view reference in the absence of ground-truth images. The term $L_{\mathrm{rad}}$ is thus a cross-stage consistency (distillation) loss that transfers the Stage-I radiance to the Stage-II physically based renderer; it is the only dense absolute-radiance reference available without images, and Stage II additionally retains the direct event-domain loss $L_{\mathrm{event}}$ so that the material decomposition remains constrained by the events rather than solely by the Stage-I reconstruction.

#### 4.3.4. Implementation Details

The method is implemented in PyTorch 2.0 on top of a differentiable 3D Gaussian rasterizer and trained using the Adam optimizer.The two stages are optimized sequentially, with Stage I trained for 30,000 iterations to reconstruct geometry and the radiance field, and Stage II for a further 10,000 iterations to recover the material parameters and environment illumination. The contrast threshold is set to C=0.2, and the global brightness anchor targets a mean rendered brightness of g=0.5. The target g=0.5 is fixed a priori as the midpoint of the normalized [0,1] intensity range, which is the natural operating point of the downstream tone-mapping and sRGB encoding; it is a predefined normalization constant applied identically to all scenes and is not selected or tuned on the evaluation metrics. The loss weights are $\lambda_{\mathrm{GW}}=0.1$ and $\lambda_{\mathrm{normal}}=0.05$ in Stage I, and $\lambda_{\mathrm{rad}}=1.0$, $\lambda_{\mathrm{GW}}=0.1$, and $\lambda_{\mathrm{TV}}=0.05$ in Stage II, with β=10 and $\lambda_{n}=0.1$ in the event-guided TV term (Equation (7)). These settings are kept fixed across all scenes unless otherwise specified.

The event streams are simulated from the rendered image sequences under an event-camera model with contrast threshold C=0.2; the event-domain loss accumulates events in fixed temporal windows aligned to the rendered frames, and the structural cue accumulates events over a short motion-compensated slice. Camera poses and intrinsics are taken directly from the dataset transforms, with no pose estimation. Both stages are optimized with Adam; the Gaussians are initialized from a random point cloud (no structure-from-motion or RGB initialization) and grown with the standard 3DGS gradient-based clone/split densification and opacity-reset schedule (opacity learning rate 0.025). Inputs are rendered at 800×800, and a full scene converges to on the order of $10^{5}$–$10^{6}$ Gaussians in a few hours on the single NVIDIA A5000, with peak GPU memory well under 24 GB.

## 5. Experiments

In this section, we evaluate the proposed event-only decomposition framework on public datasets. We assess surface normal estimation, base-color recovery, and rendered-image quality against event-input baselines that first reconstruct images from events and then apply inverse rendering, and we conduct ablation studies to isolate the contribution of each proposed component.

### 5.1. Experimental Setup

We describe the datasets, evaluation metrics, and baselines used throughout our experiments.

#### 5.1.1. Datasets and Metrics

We build our evaluation dataset upon the NeRF Synthetic dataset [3], and obtain the corresponding event data from two prior works, EventID [5] and Robust e-NeRF [29], which provide event streams synthesized from the rendered image sequences under an event camera model. We use seven scenes (chair, drums, ficus, hotdog, lego, materials, and mic), each with 400 training views and 200 test views at 800×800 resolution. The event streams are simulated from the rendered image sequences under an event-camera model, and no real-sensor recordings are used for the quantitative evaluation; each stream spans the full training trajectory of its scene. For the rendered image and base color we report PSNR [36], SSIM [36], and LPIPS [37]; for surface normals we report the mean angular error (MAE) [38].

#### 5.1.2. Baselines

To the best of our knowledge, no existing method performs geometry and material decomposition directly from event streams alone. We therefore construct event-input baselines by first converting the event stream into images and then applying an inverse-rendering pipeline. Images are reconstructed from events with an event-to-video method (E2VID [30]) and with an event-based 3D Gaussian reconstruction (Event-3DGS [39]), and are then fed into inverse-rendering methods for decomposition: GS-IR [1], GShader [10], and GI-GS [40]. We additionally consider EventID [5] as an image-and-event decomposition baseline. Because EventID requires a conventional image as its primary appearance input, we supply it with the E2VID event-to-video reconstruction, yielding an E2VID + EventID pipeline. Averaged over the seven scenes, this pipeline attains a normal MAE of $59.12^{\circ }$ and base-color/rendered PSNR of 19.94/19.39 dB, remaining below our event-only result ($28.93^{\circ }$, 22.31/22.11 dB) and consistent with the other reconstruct-then-decompose baselines.

For fairness, all baselines are given the same camera poses, intrinsics, and illumination assumptions as our method, are trained for the same number of iterations, and use the same per-method hyperparameter budget (each baseline’s own recommended defaults); the only intended difference is the input modality, that is, events for our method versus images reconstructed from the same events for the baselines. We additionally report an image-input reference that keeps our pipeline unchanged and only replaces the input with clean, well-exposed RGB images. Because events fire only when the brightness change exceeds a contrast threshold, they inherently carry less appearance information than clean images; the resulting reference attains a normal MAE of $20.18^{\circ }$ and base-color/rendered PSNR of 23.43/24.48 dB, and the gap to our event-only result reflects the appearance information lost at the event threshold rather than a limitation of the decomposition itself.

### 5.2. Comparison with State-of-the-Art Methods

We evaluate scene decomposition on a public object dataset against the event-input baselines described above. As reported in Table 1, our event-only method attains the best average score on all three tasks: the mean normal MAE drops to $28.93^{\circ }$, against $39.33^{\circ }$ for the strongest baseline (E2VID + GShader [10,30]) and above $50^{\circ }$ for the GS-IR [1]-based reconstructions, while the base-color PSNR reaches 22.31 dB against values between 18.98 and 21.03 dB and the LPIPS is the lowest on both base color and the rendered image. This gap comes from the intermediate image-recovery step: the E2VID [30] reconstructions are the weakest starting point, as their blur and inconsistent shading are baked into the estimated geometry and material, and although the Event-3DGS [39] reconstructions are sharper, a per-frame reconstruction does not keep colors and gradients consistent across views, so decomposing directly in the event domain avoids these errors. To assess robustness across scene types, we also report the across-scene standard deviation for each method, computed from the per-scene values in Table 1. For our method the normal MAE is $28.93\pm 6.28^{\circ }$, the base-color PSNR 22.31±2.74 dB, and the rendered-image PSNR 22.11±2.10 dB; these standard deviations are comparable to or smaller than those of the baselines, indicating that the improvement is consistent across scenes rather than driven by a few. The spread tracks scene difficulty, being smallest on scenes with clear boundaries and moderate specularity and largest on thin or highly specular geometry such as ficus and materials. Among the baselines, Event-3DGS + GS-IR uses the same GS-IR inverse-rendering backend as our method and differs only in the input modality (an event-reconstructed image versus our direct event-domain input); the comparison against it therefore isolates the benefit of retaining event-domain constraints during decomposition rather than any difference in the decomposition backend.

**Figure 3:**
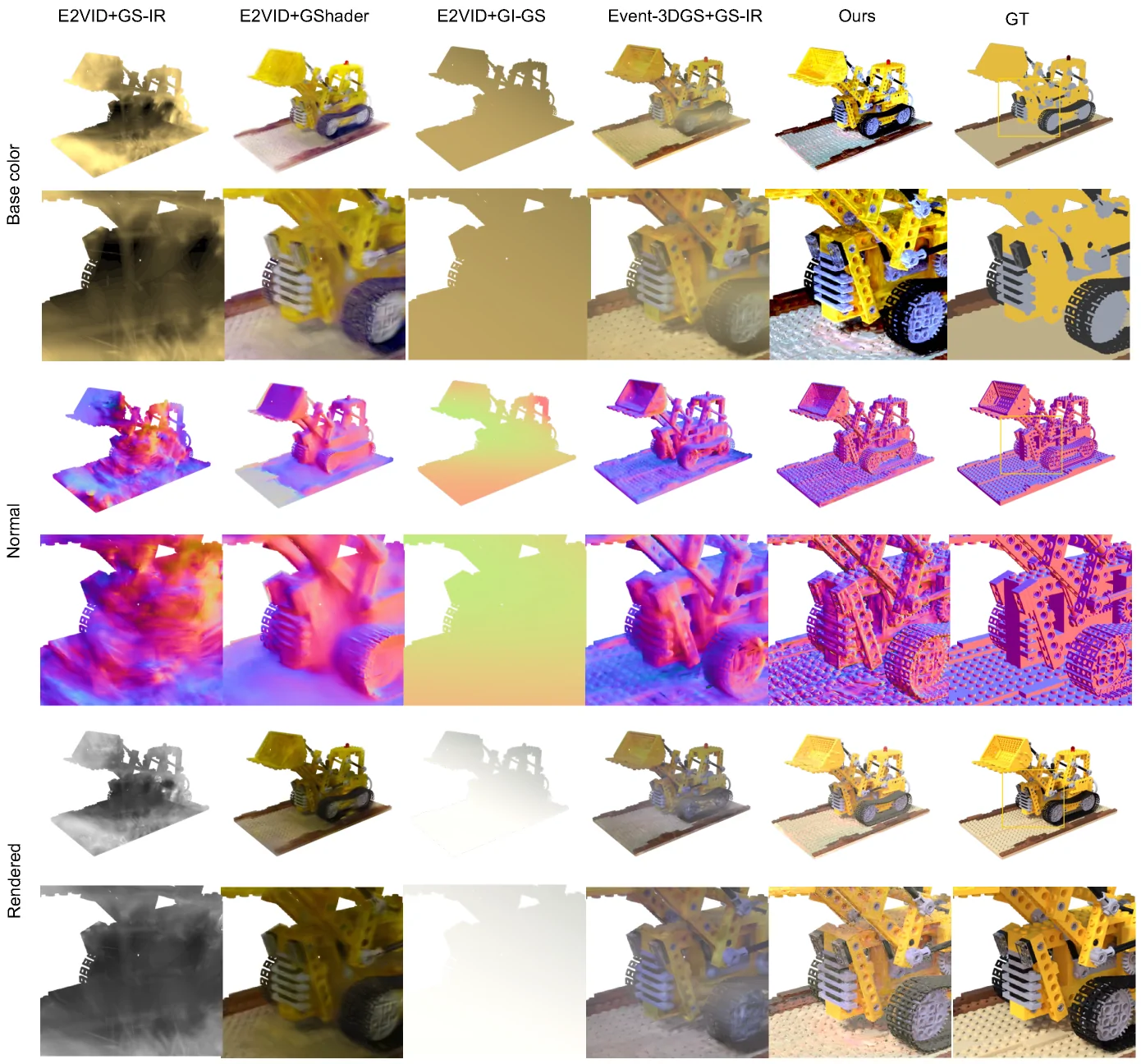
Qualitative comparison with event-input baselines on two example scenes. The columns correspond to E2VID + GS-IR [1,30], E2VID + GShader [10,30], E2VID + GI-GS [30,40], Event-3DGS + GS-IR [1,39], our event-only method, and the ground truth. For each scene, we show the recovered base color, surface normals, and rendered image (rows). The panels are enlarged and augmented with zoomed-in inset crops (highlighted boxes) so that the differences in boundary sharpness, color cast, and smooth-region cleanliness are legible at the printed size.

Figure 3 shows the same behavior qualitatively: the image-recovery baselines produce visible color casts and normals that are either noisy or over-smoothed, whereas our decomposition recovers cleaner geometry and a base color closer to the ground truth. The reason is that the reconstructed frames carry per-frame artifacts that propagate into the decomposition, while operating directly on events preserves sharp boundaries and a stable color space.

**Figure 4:**
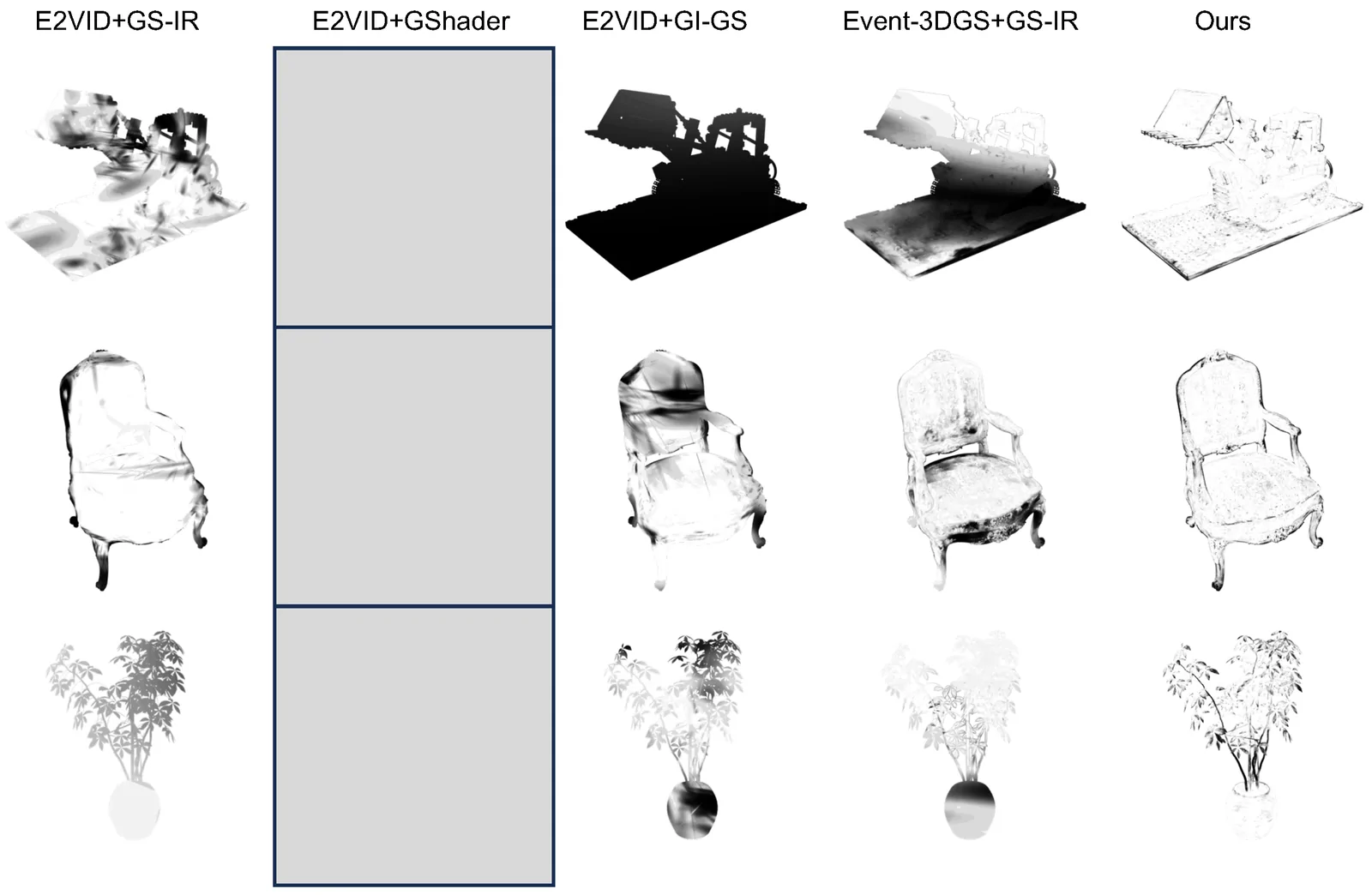
Recovered roughness maps across the event-based baselines (E2VID + GS-IR, E2VID + GShader, E2VID + GI-GS, Event-3DGS + GS-IR) and our method on three scenes. The E2VID + GShader column is left blank because that baseline does not output a roughness map.

**Figure 5:**
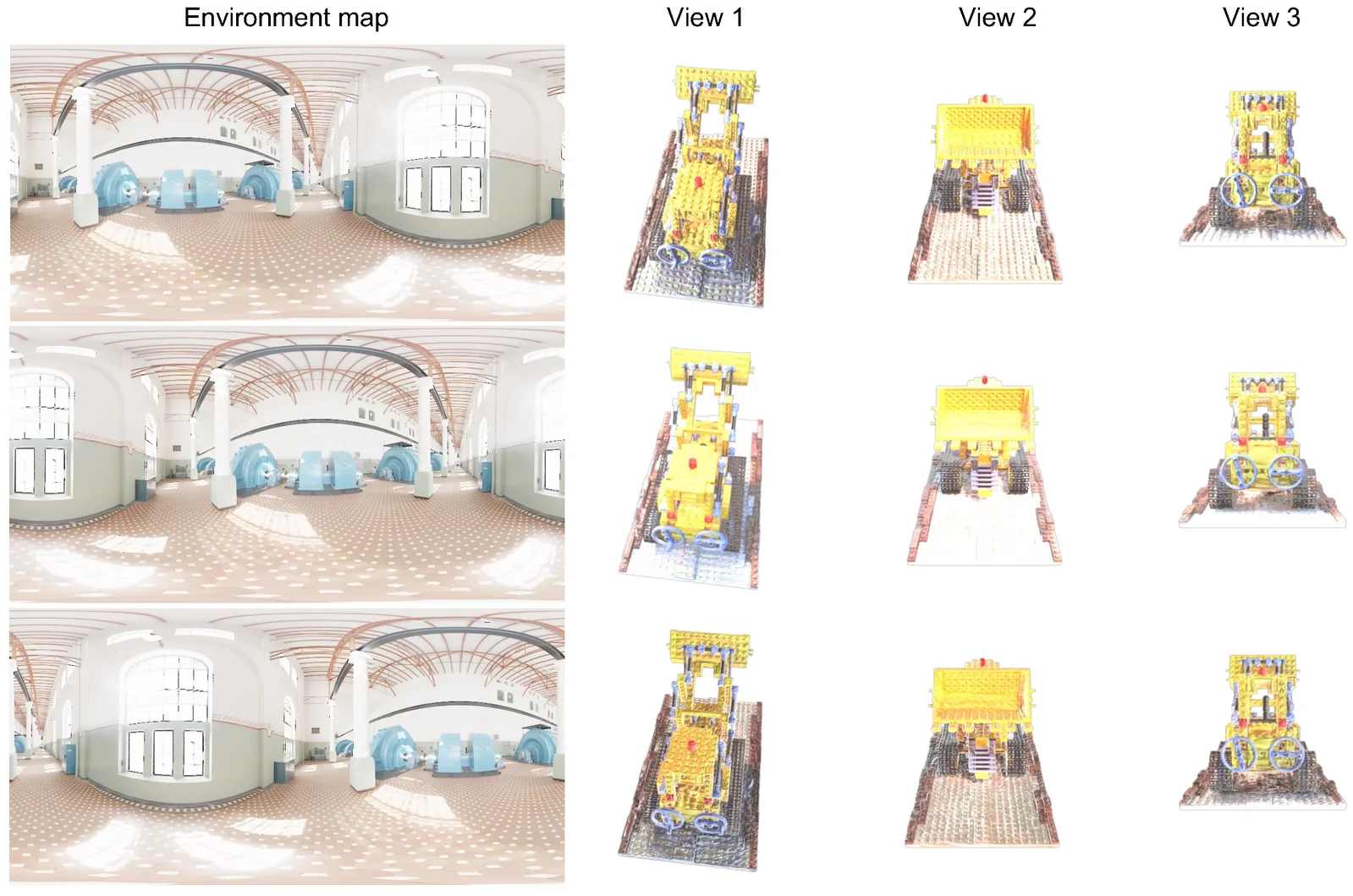
Relighting: the geometry and material recovered from events alone, re-shaded under a novel environment map rotated to three illumination directions (one per row) and shown from three test views (columns).

We quantitatively evaluate the components with reliable ground truth, namely surface normals, base color, and rendered-image quality; roughness and metallicity are recovered but not quantitatively benchmarked, as discussed in the Limitations Section. As a qualitative illustration of the material decomposition, Figure 4 compares the recovered roughness maps across the event-based baselines and our method, and Figure 5 shows a relighting example in which the geometry and material recovered from events alone are re-shaded under a novel environment map.

### 5.3. Evaluation of the Proposed Components

We further validate the two cues that our framework extracts from event streams (Section 4.2): the brightness-anchoring prior, which resolves the global brightness-scale ambiguity of the differential event signal, and the structural cue, which exploits the edge-sensitive nature of event firing to guide decomposition. We evaluate the effect of each component in turn.

#### 5.3.1. Effect of Brightness Anchoring

**Figure 6:**
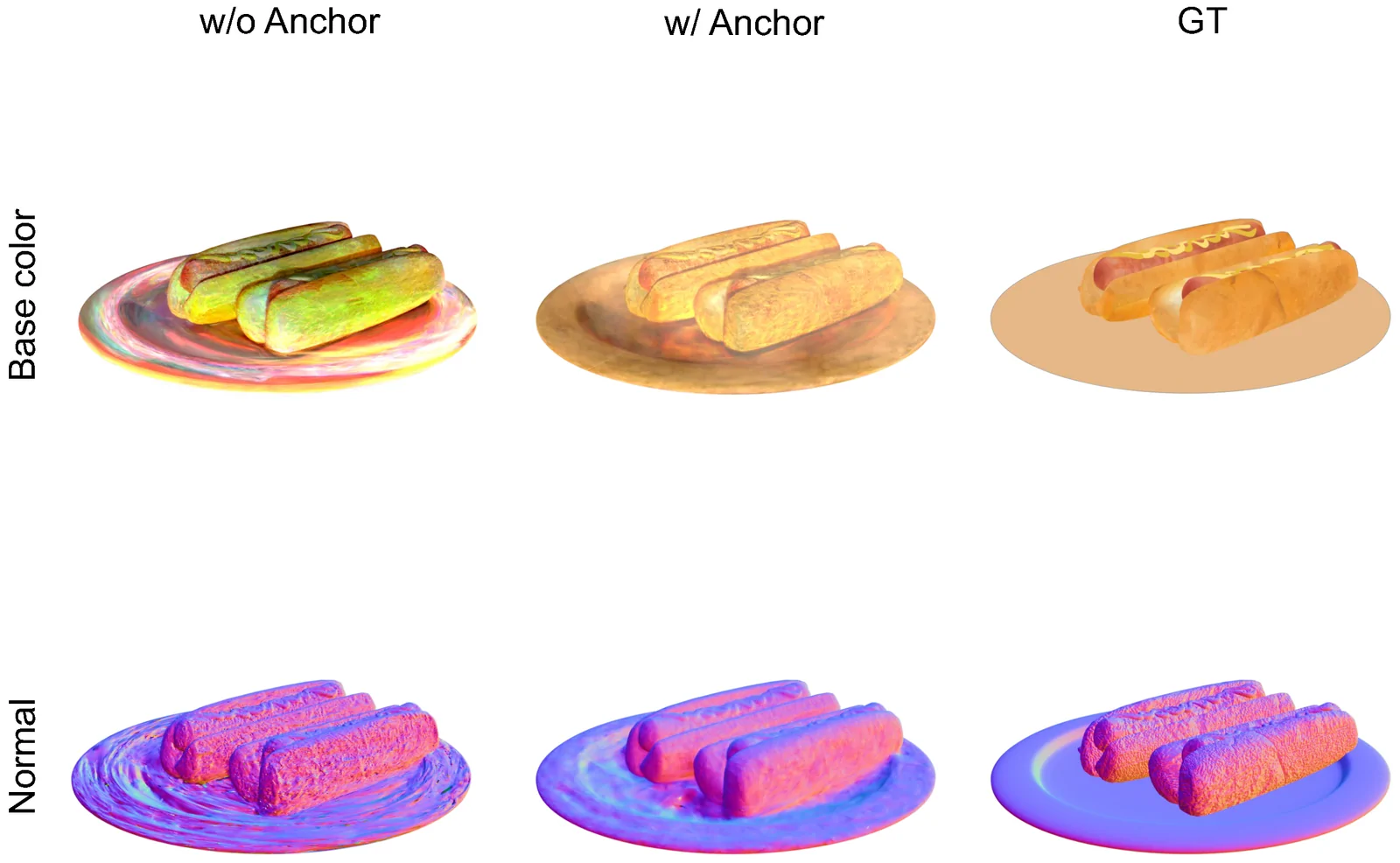
Effect of brightness anchoring from events. Without the brightness-anchoring prior, the recovered base color absorbs the unconstrained global scale and exhibits severe shifts, while enabling the prior fixes the global brightness scale and produces base color with substantially reduced color shift.

Since event streams record only differential brightness changes and carry no absolute brightness, the brightness-anchoring prior (Section 4.2) is essential for constraining the reconstructed radiance to a physically plausible global brightness scale. As shown in Figure 6, without brightness anchoring the free global scale is absorbed into the recovered base color, which drifts toward degenerate, arbitrarily scaled solutions, whereas enabling the anchoring prior yields base color that is consistent with the ground truth.

Table 2 quantifies this effect: brightness anchoring improves base-color PSNR by 3.13 dB and reduces the normal MAE by $7.94^{\circ }$. Fixing the global brightness scale therefore helps geometry optimization as well, since a stably scaled radiance field provides the material stage with a more consistent appearance cue.

#### 5.3.2. Effect of Event-Derived Structural Cues

We next evaluate the structural cue introduced in Section 4.2, in which the event-derived edge map guides a total variation (TV) regularization on the decomposition outputs (Equation (7)). Since events fire predominantly at object boundaries, depth discontinuities, and material transitions, this structural cue is expected to preserve sharp discontinuities at genuine edges while suppressing high-frequency artifacts in smooth regions. To isolate its effect, we compare the full model against a variant that removes the structural cue (i.e., without the event-guided TV term), reporting surface normal accuracy, recovered base color, and the rendered image in Table 2.

**Figure 7:**
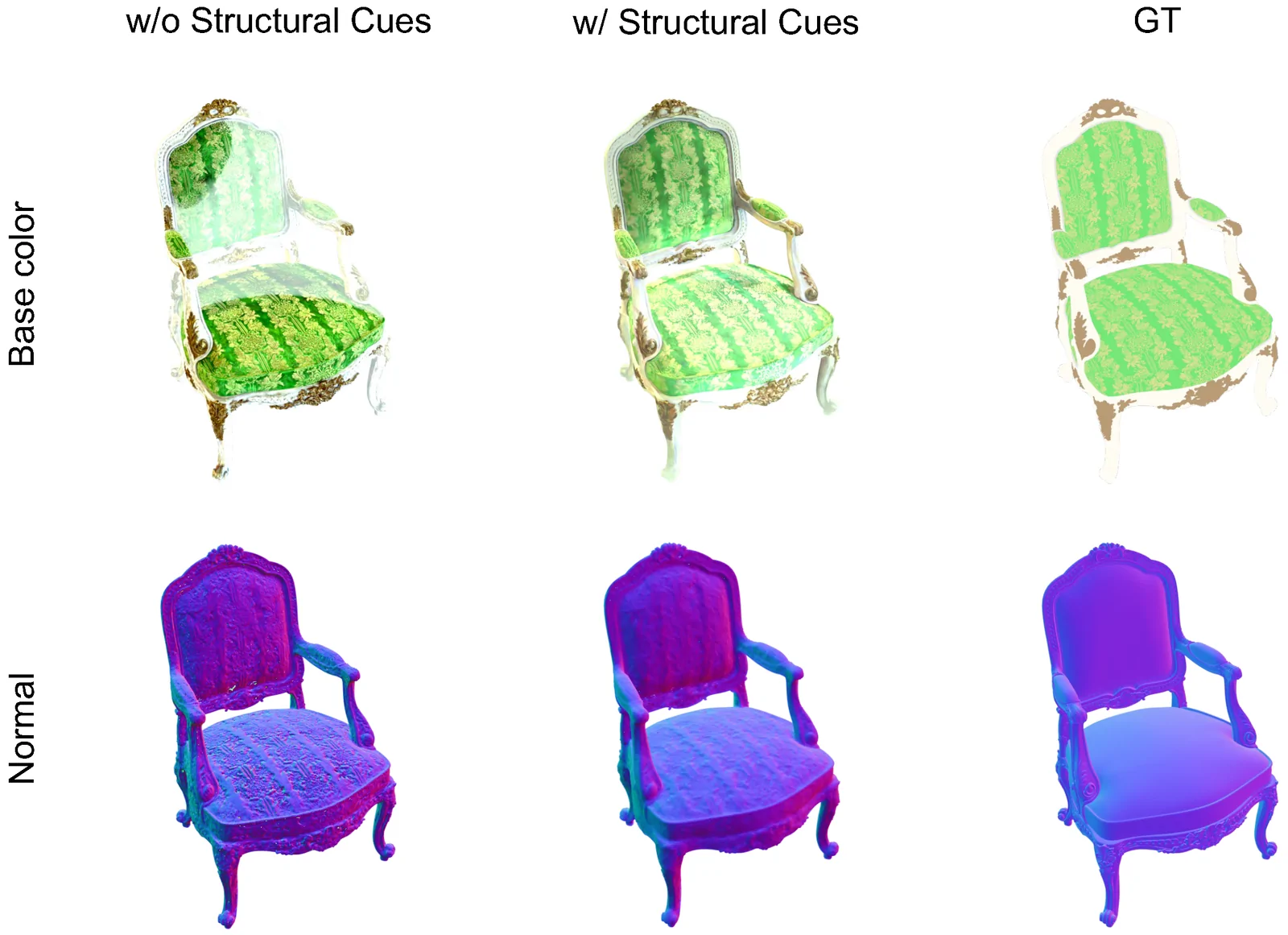
Qualitative effect of the event-derived structural cue. Compared with the variant without the event-guided total variation regularization, our full model suppresses high-frequency artifacts in smooth regions while preserving sharp discontinuities at genuine object boundaries indicated by events.

As shown in Table 2, removing the structural cue increases the normal MAE by $6.77^{\circ }$ and lowers base-color PSNR, with the largest effect on geometry. Because the event-guided regularization relaxes smoothing only near event-dense boundaries, its benefit concentrates on sharpening geometry rather than on uniformly changing appearance. This is consistent with the design of the cue: the event-guided TV term relaxes smoothness only at event-dense boundaries and enforces it elsewhere, so it preserves sharp discontinuities at genuine edges while suppressing high-frequency artifacts in smooth regions. A qualitative comparison is shown in Figure 7, where the structural cue produces cleaner surfaces in smooth regions while preserving sharp boundaries and geometric detail at object edges.

### 5.4. Ablation Study

This section studies the effect of the event contrast threshold *C*, which is the key parameter governing how event observations are generated from the underlying radiance, and additionally examines the Stage-I normal-consistency loss and the sensitivity of the decomposition to the principal loss weights.

#### 5.4.1. Sensitivity to the Event Contrast Threshold

The contrast threshold *C* sets the minimum log-radiance change required to trigger an event (Equation (2)), and thus controls how much scene information the event stream carries. A threshold that is too small lets sensor noise enter the supervision as if it were real signal, whereas a threshold that is too large suppresses the events triggered by genuine contrast changes, leaving low-contrast textures and subtle shading variations unobserved. Here *C* is the threshold used in the training-supervision event loss; the simulated event streams are held fixed, so this sweep measures robustness to a mismatch between the assumed and the true threshold rather than re-simulating the events. Because *C* scales the event-domain residual globally, the radiance optimization absorbs it, and the method does not require knowing the true sensor contrast threshold precisely. Since our framework recovers geometry and material purely from events, the amount of detail preserved by the event stream directly bounds the achievable decomposition quality. Table 3 sweeps *C* over the range observed in practice, holding all other settings at their default values and reporting surface normal accuracy and base-color recovery.

As shown in Table 3, varying the contrast threshold *C* over the tested range has only a minor effect on the decomposition quality on the evaluated dataset, and all metrics remain largely stable. The method is thus not sensitive to the exact value of *C* within this range, as the event stream still preserves the dominant geometric and color cues. We therefore set C=0.2 in all other experiments.

#### 5.4.2. Effect of the Stage-I Normal-Consistency Loss

We assess the Stage-I normal-consistency loss $L_{\mathrm{normal}}$ by retraining the complete pipeline with it disabled ($\lambda_{\mathrm{normal}}=0$) while keeping all other settings unchanged. As reported in Table 4, removing the term degrades the recovered surface normals, with the mean angular error rising from $28.93^{\circ }$ to $33.45^{\circ }$, while the rendered image is essentially unchanged (PSNR 22.11 vs. 21.98 dB). The normal-consistency term therefore acts as a geometry regularizer that improves surface-normal accuracy at negligible cost to the rendered appearance, which is why we retain it.

#### 5.4.3. Sensitivity to the Loss Weights

We further sweep the two principal loss weights one at a time, the brightness-anchor weight $\lambda_{\mathrm{GW}}$ and the event-guided TV weight $\lambda_{n}$, holding all other settings at their default values (Table 5). Across the tested ranges the rendered PSNR varies by less than 0.3 dB for $\lambda_{\mathrm{GW}}$ and by less than 0.2 dB for $\lambda_{n}$, so the decomposition is not sensitive to the exact weight values within these ranges. We report the selected default values together with this empirical sensitivity.

#### 5.4.4. Sensitivity to the Brightness-Anchor Target

We evaluate the sensitivity of the global brightness anchor to its target value *g* by sweeping g∈{0.3,0.5,0.7} with all other settings fixed (Table 6). The default g=0.5 attains the best geometry and rendered-image quality; shifting the target to 0.3 or 0.7 biases the reconstructed brightness and degrades both the surface-normal accuracy and the rendered PSNR. Because the anchor constrains only the scalar mean brightness while the per-channel color variation is fixed by the RGGB event observations, scenes dominated by a single hue or by colored illumination do not directly violate it.

#### 5.4.5. Independent Geometry Evaluation

The Stage-I normal-consistency loss compares rendered normals with pseudo-normals derived from the model’s own depth and is therefore an internal regularizer rather than independent geometry supervision. To assess geometry against an external reference, we additionally compute the foreground depth error of the recovered geometry against the dataset ground-truth depth maps. Averaged over the seven scenes, the recovered geometry attains a mean absolute depth error of 0.037 scene units and a mean absolute-relative error of 1.0%, confirming that the recovered surfaces are accurate and not merely internally self-consistent.

#### 5.4.6. Random-Seed Stability and Sequential Optimization

Re-running the full two-stage pipeline from three independent random seeds on three representative scenes changes the rendered PSNR by only 0.05–0.43 dB (Table 7), far below the gaps between methods in Table 1, so the reported results are not an artifact of a favorable seed. We also compare the two-stage design against a single-pass joint optimization of geometry and material: the joint variant does not improve on the two-stage pipeline (rendered PSNR 24.21 vs. 24.77 dB on the chair scene), consistent with the ill-posedness of jointly optimizing tightly coupled geometry and material from events that carry no absolute appearance.

#### 5.4.7. Effect of the Cross-Stage Radiance Loss

The Stage-II radiance loss $L_{\mathrm{rad}}$ (Equation (13)) transfers the Stage-I radiance to the physically based renderer and is the only dense absolute-radiance reference available without images. Table 8 isolates its effect by resuming Stage II from the same Stage-I checkpoint with and without the term. Removing $L_{\mathrm{rad}}$ lowers the rendered PSNR by about 4.9 dB, confirming that this reference is necessary for a stable Stage-II decomposition. For reference, the same pipeline supervised with clean, well-exposed RGB images attains a higher rendered PSNR, quantifying the residual effect of imperfect Stage-I radiance.

#### 5.4.8. Robustness to Pose Error

**Figure 8:**
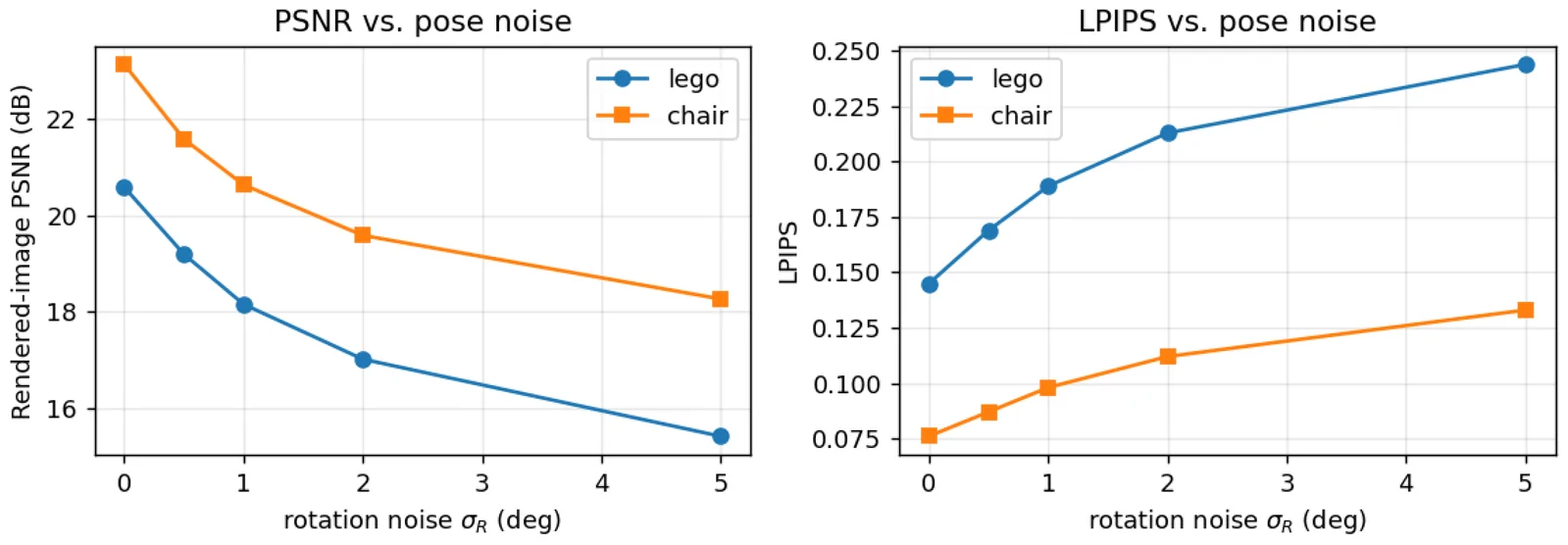
Rendered PSNR and LPIPS versus pose noise on lego and chair; the decomposition degrades smoothly and monotonically.

Because the camera poses may in practice be estimated from the events rather than given, we characterize the robustness of the decomposition to pose error by injecting controlled Gaussian noise into the input poses (rotation $\sigma_{R}$, translation $\sigma_{t}$) and re-evaluating (Table 9, Figure 8). The recovery degrades gracefully and monotonically: a realistic $0.5^{\circ }$/0.005 perturbation lowers the rendered PSNR by only 1.5–1.7 dB, and a $5^{\circ }$/0.05 error is needed to lose 5–6 dB, indicating tolerance to the pose error typical of event-based trackers rather than a sharp failure.

## 6. Limitations and Future Work

### 6.1. Limitations

Under normal, well-exposed imaging conditions, our event-only method does not surpass image-based decomposition methods. The reason is intrinsic to the sensor: an event camera reports only brightness changes above a fixed contrast threshold, so low-contrast textures and subtle intensity variations trigger no events and are not observed, and the fine appearance detail that clean RGB images capture directly is unavailable to an event-only approach.

Our experiments use event streams simulated from synthetic multi-view scenes, and the reported results should therefore be interpreted within this setting. Physical event sensors exhibit effects that are absent or only partially represented in the evaluated sequences, including background activity, hot pixels, pixel- and polarity-dependent contrast thresholds, refractory effects, finite bandwidth, dropped events, timestamp noise, and pixel-response non-uniformity. These effects may perturb the event-domain photometric loss and reduce the sharpness and reliability of the motion-compensated structural cue. Robust event losses, explicit sensor calibration, and jointly estimated or learned threshold parameters are possible mitigations. Validation on recordings from a physical event camera remains necessary and is a primary direction for future work.

**Figure 9:**
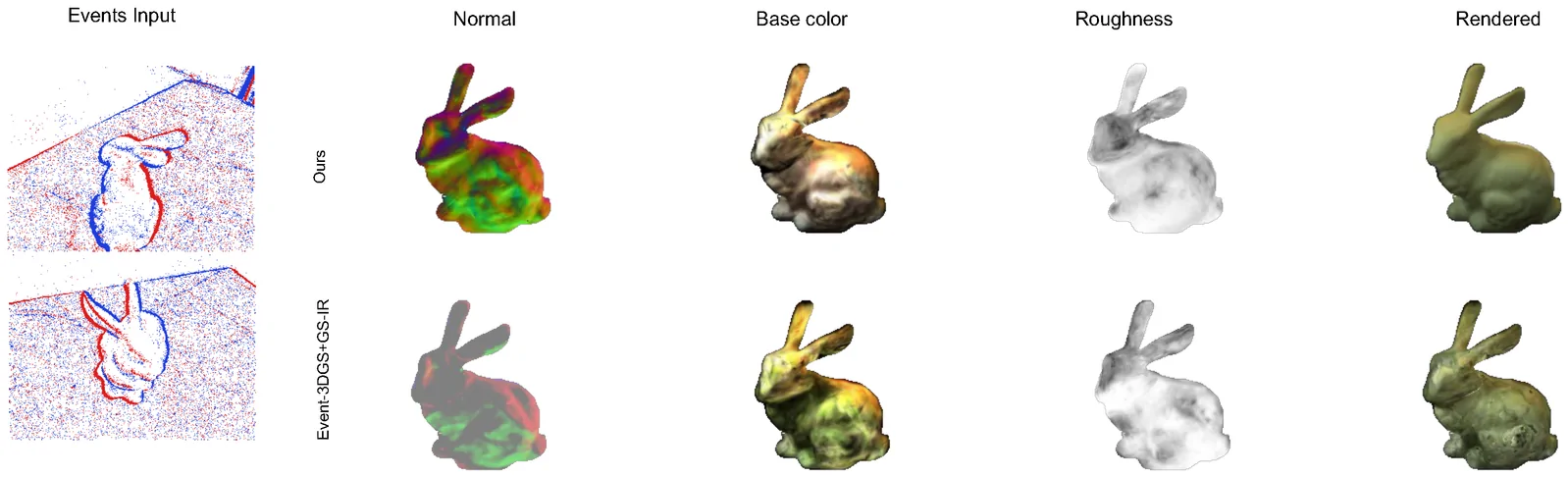
Qualitative decomposition on real event-camera data captured with a Color-DAVIS346 event camera. Columns: Accumulated events (blue +, red −), recovered surface normals, base color, roughness, and rendered image; rows compare our method with the Event-3DGS + GS-IR baseline.

As a preliminary step toward real-sensor validation, we additionally apply the trained pipeline to a real event-camera sequence captured with a DAVIS sensor (Figure 9). Because real captures provide no ground-truth geometry or material, this demonstration is qualitative: the method recovers coherent surface normals, a plausible base color, and a rendered image directly from the real event stream, and remains competitive with the Event-3DGS + GS-IR baseline on the same sequence. Full quantitative benchmarking on real recordings remains future work.

Finally, the estimated roughness is weakly constrained by events: its foreground mean is close to the upper bound across the evaluated scenes (approximately 0.97–1.00 on the normalized [0,1] scale), and the resulting maps do not resolve fine gloss variation. We therefore treat roughness as recovered but not quantitatively benchmarked, and metallicity is fixed under a dielectric-material assumption; this reflects a limitation of the present data, reflectance formulation, and supervision rather than an inherent inability of event measurements to encode specular information. The displayed material maps use the original normalized values without contrast-based renormalization.

The event observations also do not uniquely determine the global per-channel radiance scales. The event generation model is invariant to an independent constant multiplicative factor on each color channel, and the brightness anchor constrains only the overall mean brightness, so the absolute inter-channel color balance is a gauge freedom that events cannot resolve. In the evaluated synthetic scenes the illumination is approximately neutral, which keeps the recovered base color close to the reference, but recovering the absolute color balance under strongly colored illumination would require an external radiometric or white-balance reference.

Finally, the event-domain loss treats a pixel with no recorded event as having a zero accumulated count. Physically, a zero count indicates only that the log-radiance change remained below the contrast threshold during that interval, not that the change is exactly zero. Assigning a zero target to such a pixel therefore replaces this threshold-bounded observation with a point-valued optimization surrogate, which we identify as a limitation of the present event-domain loss.

### 6.2. Future Work

A natural direction to address this limitation is to bridge the gap between event-only and image-based decomposition by combining event-derived cues with sparse or intermittent image supervision. In such a hybrid formulation, the events would continue to provide robust structural and color cues under degradation, while a small number of image observations could supply the absolute intensity and fine appearance detail that fall below the event contrast threshold. This would allow the fine detail lost in the event-only setting to be recovered while retaining the robustness of events under challenging capture conditions.

## 7. Conclusions

We introduced an event-only framework for recovering scene geometry and material with 3D Gaussian Splatting. A brightness-anchoring prior constrains the underdetermined global brightness scale of the reconstructed radiance field, and motion-compensated event edges guide boundary-aware regularization. On public datasets, the method achieves the best average performance among pipelines that first reconstruct images and then apply inverse rendering. On the seven evaluated synthetic scenes, it reaches a mean surface-normal angular error of $28.93^{\circ }$, a base-color PSNR/SSIM/LPIPS of 22.31 dB/0.924/0.117, and a novel-view rendering PSNR/SSIM/LPIPS of 22.11 dB/0.902/0.105, the best average scores among the compared event-input methods. Its main limitation is the loss of low-contrast appearance information that does not trigger events, and validation on real event data remains future work.

In summary, the main findings of this study are as follows:

1. We developed a 3D Gaussian Splatting framework for estimating scene geometry and base color from simulated event streams without using RGB images as appearance input; the framework assumes known camera poses and intrinsics.
2. We formulated an event-domain reflectance model that incorporates visibility and indirect illumination, extending event-based decomposition from an isolated-object formulation to a scene-level representation.
3. Global brightness anchoring and event-guided structural regularization provide complementary constraints for the differential and spatially sparse event measurements; the ablation results show measurable changes in base-color and geometry metrics when either component is removed.
4. On the seven evaluated synthetic scenes, the proposed method achieves the best average performance among the compared event-input pipelines that first reconstruct images and then apply inverse rendering.

The current findings are limited to simulated event streams, and validation on recordings from physical event cameras remains necessary. In addition, the current event measurements provide insufficient constraints for estimating fine roughness variations, while metallicity is fixed under the dielectric-material assumption; accordingly, we do not claim general recovery of spatially varying physically based material parameters beyond geometry and base color.

## Figures and Tables

**Table 1:** Quantitative comparison with event-input methods on a public object dataset (7 scenes, 200 test poses per scene), reported per scene. Best per scene and metric in **bold**, second best underlined. PSNR: Peak signal-to-noise ratio; SSIM: structural similarity index measure; LPIPS: learned perceptual image patch similarity; MAE: mean angular error. Upward (↑) and downward (↓) arrows indicate that higher and lower values are better, respectively.

Method	Scene	Normal	Base Color			Rendered Image		
		**MAE** (°) ↓	**PSNR** ↑	**SSIM** ↑	**LPIPS** ↓	**PSNR** ↑	**SSIM** ↑	**LPIPS** ↓
E2VID + GI-GS [30,40]	chair	55.15	19.94	0.894	0.151	18.57	0.861	0.162
	drums	47.63	17.49	0.867	0.167	17.52	0.866	0.164
	ficus	73.28	26.99	0.975	0.043	20.03	0.928	0.102
	hotdog	44.34	19.83	0.901	0.177	16.93	0.879	0.196
	lego	58.12	19.33	0.851	0.186	20.08	0.871	0.141
	materials	64.85	19.42	0.887	0.171	15.38	0.832	0.212
	mic	48.14	22.95	**0.960**	0.054	21.55	0.936	0.084
	Mean	55.93	20.85	0.905	0.136	18.58	0.882	0.152
E2VID + GShader [10,30]	chair	36.64	20.40	0.896	0.146	16.65	0.854	0.139
	drums	36.34	**18.62**	0.881	0.144	19.23	0.875	0.144
	ficus	44.89	26.62	0.967	0.044	22.87	0.934	0.079
	hotdog	37.97	19.99	0.898	0.186	18.08	0.878	0.157
	lego	42.63	19.25	0.850	0.216	17.54	0.821	0.182
	materials	43.99	18.32	0.872	0.190	18.36	**0.846**	0.192
	mic	32.84	**23.98**	0.959	0.066	24.78	0.948	0.067
	Mean	39.33	21.03	0.903	0.142	19.64	0.879	0.137
E2VID + GS-IR [1,30]	chair	64.08	14.75	0.866	0.176	15.50	0.844	0.190
	drums	57.85	16.00	0.849	0.189	16.51	0.852	0.186
	ficus	77.94	26.11	0.970	0.047	17.42	0.905	0.118
	hotdog	57.53	17.73	0.871	0.200	15.32	0.843	0.242
	lego	55.56	16.28	0.855	0.215	15.13	0.783	0.267
	materials	70.39	19.53	0.923	0.167	14.77	0.818	0.210
	mic	53.46	22.47	0.959	**0.044**	18.00	0.917	0.093
	Mean	62.40	18.98	0.899	0.148	16.09	0.852	0.187
Event-3DGS + GS-IR [1,39]	chair	55.35	19.53	0.882	0.116	22.47	0.909	0.118
	drums	45.53	17.15	0.826	0.153	19.64	0.875	0.122
	ficus	70.03	26.55	0.953	0.045	18.17	0.923	0.100
	hotdog	53.95	15.88	0.817	0.230	18.17	0.871	0.174
	lego	50.61	18.18	0.794	0.200	18.70	0.856	0.162
	materials	66.94	15.78	0.786	0.204	16.39	0.830	0.190
	mic	30.60	21.73	0.911	0.092	**25.18**	0.946	0.061
	Mean	53.29	19.26	0.853	0.149	19.82	0.887	0.132
Ours	chair	**27.47**	**23.35**	**0.923**	**0.108**	**24.77**	**0.925**	**0.074**
	drums	**25.89**	18.40	**0.885**	**0.130**	**20.06**	**0.893**	**0.110**
	ficus	**40.24**	**27.36**	**0.977**	**0.039**	**23.51**	**0.939**	**0.072**
	hotdog	**19.16**	**22.41**	**0.919**	**0.143**	**19.77**	**0.905**	**0.142**
	lego	**29.12**	**20.79**	**0.879**	**0.179**	**22.75**	**0.894**	**0.103**
	materials	**34.67**	**19.92**	**0.926**	**0.163**	**19.54**	0.803	**0.176**
	mic	**25.97**	23.92	**0.960**	0.058	24.36	**0.952**	**0.057**
	Mean	**28.93**	**22.31**	**0.924**	**0.117**	**22.11**	**0.902**	**0.105**

**Table 2:** Ablation of the two proposed modules, averaged over the public dataset. “Ours” denotes the full model, and each other row removes one module. Best per column in **bold**. PSNR: Peak signal-to-noise ratio; SSIM: structural similarity index measure; LPIPS: learned perceptual image patch similarity; MAE: mean angular error. Upward (↑) and downward (↓) arrows indicate that higher and lower values are better, respectively.

Module	Normal	Base Color			Rendered Image		
	**MAE** (**°**) **↓**	**PSNR ↑**	**SSIM ↑**	**LPIPS ↓**	**PSNR ↑**	**SSIM ↑**	**LPIPS ↓**
w/o Brightness Anchoring	36.87	19.18	0.854	0.147	20.51	0.886	0.113
w/o Structural Cues	35.70	20.69	0.886	0.136	21.52	0.894	0.106
Ours	**28.93**	**22.31**	**0.924**	**0.117**	**22.11**	**0.902**	**0.105**

**Table 3:** Effect of the event contrast threshold *C* on decomposition quality, averaged over the public dataset. PSNR: Peak signal-to-noise ratio; SSIM: structural similarity index measure; LPIPS: learned perceptual image patch similarity; MAE: mean angular error. Upward (↑) and downward (↓) arrows indicate that higher and lower values are better, respectively.

Contrast Threshold *C*	Normal	Base Color			Rendered Image		
	**MAE** (°) ↓	**PSNR** ↑	**SSIM** ↑	**LPIPS** ↓	**PSNR** ↑	**SSIM** ↑	**LPIPS** ↓
0.1	29.12	22.23	0.893	0.126	22.04	0.898	0.109
0.2	28.93	22.31	0.924	0.117	22.11	0.902	0.105
0.3	29.31	21.89	0.894	0.116	21.97	0.901	0.115

**Table 4:** Effect of the Stage-I normal-consistency loss $L_{\mathrm{normal}}$, averaged over the public dataset. Upward (↑) and downward (↓) arrows indicate that higher and lower values are better, respectively.

Setting	Normal	Rendered Image		
	**MAE (°)** ↓	**PSNR** ↑	**SSIM** ↑	**LPIPS** ↓
With $L_{\mathrm{normal}}$ (Ours)	28.93	22.11	0.902	0.105
Without ($\lambda_{\mathrm{normal}}$ = 0)	33.45	21.98	0.899	0.109

**Table 5:** Sensitivity to the brightness-anchor weight $\lambda_{\mathrm{GW}}$ and the event-guided TV weight $\lambda_{n}$, averaged over the public dataset. “Ours” marks the default setting. Upward (↑) and downward (↓) arrows indicate that higher and lower values are better, respectively.

Group	Setting	Normal	Base Color			Rendered Image		
		**MAE (°)** ↓	**PSNR** ↑	**SSIM** ↑	**LPIPS** ↓	**PSNR** ↑	**SSIM** ↑	**LPIPS** ↓
Brightness anchor $\lambda_{\mathrm{GW}}$	0.1 (Ours)	28.93	22.31	0.924	0.117	22.11	0.902	0.105
	0.2	28.88	22.12	0.910	0.119	22.03	0.900	0.106
	0.3	28.91	22.16	0.916	0.117	22.04	0.891	0.103
Normal TV $\lambda_{n}$	0.1 (Ours)	28.93	22.31	0.924	0.117	22.11	0.902	0.105
	0.2	29.01	22.28	0.921	0.116	22.08	0.901	0.104
	0.3	29.41	22.14	0.905	0.121	21.93	0.884	0.110

**Table 6:** Sensitivity of the global brightness anchor to the target *g*, averaged over the public dataset. “default” marks the setting used in all other experiments. Upward (↑) and downward (↓) arrows indicate that higher and lower values are better, respectively.

Brightness Target *g*	Normal	Base Color			Rendered Image		
	**MAE (°)** ↓	**PSNR** ↑	**SSIM** ↑	**LPIPS** ↓	**PSNR** ↑	**SSIM** ↑	**LPIPS** ↓
0.3	34.90	18.29	0.861	0.138	20.05	0.879	0.116
0.5 (default)	28.93	22.31	0.924	0.117	22.11	0.902	0.105
0.7	41.24	17.91	0.879	0.142	19.93	0.881	0.119

**Table 7:** Random-seed stability: the full two-stage pipeline retrained from three independent seeds on three representative scenes (rendered novel-view metrics, mean ± standard deviation). Upward (↑) and downward (↓) arrows indicate that higher and lower values are better, respectively.

Scene	#Seeds	PSNR ↑	SSIM ↑	LPIPS ↓
lego	3	22.75±0.42	0.894±0.003	0.103±0.002
chair	3	24.77±0.43	0.925±0.011	0.074±0.004
ficus	3	23.51±0.05	0.939±0.001	0.072±0.002
mean	3	23.68	0.919	0.083

**Table 8:** Effect of the cross-stage radiance loss $L_{\mathrm{rad}}$ in Stage II (seven-scene mean, rendered novel-view metrics). “Clean RGB” replaces the Stage-I reference with clean images as an upper reference. Upward (↑) and downward (↓) arrows indicate that higher and lower values are better, respectively.

Radiance Supervision in Stage II	Render PSNR ↑	Render LPIPS ↓
Without $L_{\mathrm{rad}}$	17.22	0.171
Stage-I radiance (Ours)	22.11	0.105
Clean RGB (reference)	24.48	0.102

**Table 9:** Pose-perturbation sensitivity on legoand chair (rendered novel-view metrics). $\sigma_{t}$ is in scene units (object radius ≈ 4). Upward (↑) and downward (↓) arrows indicate that higher and lower values are better, respectively.

		Lego			Chair		
$\sigma_{R}{(}^{\circ })$	$\sigma_{t}$	**PSNR** ↑	**SSIM** ↑	**LPIPS** ↓	**PSNR** ↑	**SSIM** ↑	**LPIPS** ↓
0.0	0.000	22.75	0.894	0.103	24.77	0.925	0.074
0.5	0.005	21.21	0.830	0.120	23.11	0.880	0.085
1.0	0.010	20.06	0.795	0.134	22.09	0.861	0.095
2.0	0.020	18.80	0.773	0.151	20.97	0.850	0.109
5.0	0.050	17.03	0.763	0.173	19.55	0.843	0.130

## Data Availability

This study uses publicly available datasets [3].
